# Concordance with SPIRIT-AI guidelines in reporting of randomized controlled trial protocols investigating artificial intelligence in oncology: a systematic review

**DOI:** 10.1093/oncolo/oyaf112

**Published:** 2025-05-27

**Authors:** David Chen, Emily He, Keiran Pace, Matthew Chekay, Srinivas Raman

**Affiliations:** Princess Margaret Hospital Cancer Centre, Radiation Medicine Program, Toronto, Ontario, Canada M5G 2C4; Temerty Faculty of Medicine, University of Toronto, Toronto, ON, Canada M5S 3K3; Temerty Faculty of Medicine, University of Toronto, Toronto, ON, Canada M5S 3K3; Temerty Faculty of Medicine, University of Toronto, Toronto, ON, Canada M5S 3K3; Temerty Faculty of Medicine, University of Toronto, Toronto, ON, Canada M5S 3K3; Princess Margaret Hospital Cancer Centre, Radiation Medicine Program, Toronto, Ontario, Canada M5G 2C4; Department of Radiation Oncology, University of Toronto, Toronto, ON, Canada M5T 1P5; Department of Radiation Oncology, BC Cancer Vancouver, Vancouver, BC, Canada V5Z 1M9

**Keywords:** randomized controlled trial, RCT, protocol, artificial intelligence, AI, SPIRIT, SPIRIT-AI

## Abstract

**Background:**

Artificial intelligence (AI) is a promising tool used in oncology that may be able to facilitate diagnosis, treatment planning, and patient management. Transparency and completeness of protocols of randomized controlled trials (RCT) involving AI interventions is necessary to ensure reproducibility of AI tools across diverse clinical settings. The SPIRIT 2013 and SPIRIT-AI 2020 guidelines were developed as evidence-based recommendations for complete reporting of trial protocols. However, the concordance of AI RCT protocols in oncology to SPIRIT reporting guidelines remains unknown. This systematic review evaluates the concordance of protocols of RCTs evaluating AI interventions in oncology to the SPIRIT 2013 and SPIRIT-AI 2020 reporting guidelines.

**Methods:**

A systematic search of Ovid Medline and Embase was conducted on October 22, 2024 for primary, peer-reviewed RCT protocols involving AI interventions in oncology. Eligible studies were screened in duplicate and data extraction assessed concordance to SPIRIT 2013 and SPIRIT-AI 2020 guideline items. Item-specific concordance was measured as the proportion of studies that reported the item. Average concordance was measured as the median proportion of items reported for each study.

**Results:**

Twelve RCT protocols met the inclusion criteria. The median concordance to SPIRIT 2013 guidelines was 81.92% (IQR 74.88-88.95) and the median concordance to SPIRIT-AI 2020 guidelines was 78.21% (IQR 67.21-89.20). For SPIRIT 2013 guidelines, high concordance was observed for items related to study objectives and ethics, but gaps were identified in reporting blinding procedures, participant retention, and post-trial care. For SPIRIT-AI 2020 guidelines, there remained gaps based on data quality management, performance error analysis, and accessibility of AI intervention code.

**Conclusion:**

While concordance to reporting guidelines in oncology AI RCT protocols was moderately high, critical gaps in protocol reporting persist that may hinder reproducibility and clinical implementation. Future efforts should focus on increasing awareness and reinforcement to enhance reporting quality necessary to foster the responsible integration of AI into oncology practice.

**Implications for practice**

Artificial intelligence (AI) has the potential to transform cancer care by enhancing early detection, diagnosis, and treatment planning. To effectively integrate AI tools into clinical practice, protocols of randomized controlled trials (RCTs) that assess these tools must be transparent and complete. This systematic review reveals moderate concordance of RCT protocols of AI interventions in oncology to SPIRIT-AI reporting guidelines but highlights key gaps in areas such as data quality management, blinding, and post-trial care. Addressing these gaps is essential to ensure the reproducibility, safety, and ethical deployment of AI technologies in oncology to meaningfully and reliably improve cancer patient outcomes.

## Introduction

The rapid advancement of artificial intelligence (AI) offers transformative potential in applications such as early detection, diagnosis, treatment planning, and patient management in oncology.^1^ AI-driven tools can leverage large-scale, multi-modal cancer data to provide insights that can enhance clinical decision-making and improve patient outcomes.^2,3^ Despite the enthusiasm surrounding AI applications in oncology, the complexity of these tools necessitates rigorous evaluation to ensure their safety, efficacy, and ethical deployment in clinical practice.^4,5^

Evaluations of the safety and efficacy of interventions in medicine are often conducted through gold-standard randomized controlled trials (RCTs).^6^ RCTs provide a systematic approach to assess the impact of AI interventions, that are necessary to inform meaningful practice changes.^7^ To ensure the validity and reproducibility of RCT findings, and avoid duplicative efforts, authors are encouraged to publish the associated RCT protocol. Inadequate reporting of trial protocols can lead to biases, reduce transparency, and hinder the implementation of AI-based interventions in clinical practice. Moreover, guidelines for RCT protocol reporting have varied greatly in their scope and recommendations, often failing to describe the development of the guideline, or cite empirical evidence to guide their recommendations.^8^

To address these challenges, the Standard Protocol Items: Recommendations for Interventional Trials (SPIRIT) 2013 guidelines were developed to provide a 33-item framework for the comprehensive and transparent reporting of trial protocols.^9^ Recognizing the unique challenges posed by AI interventions, the SPIRIT-AI extension was introduced in 2020 to address the specific considerations related to the design and conduct of AI-based clinical trials.^10^ The SPIRIT-AI extension includes additional guidance to ensure that key aspects such as AI system development, validation, and integration into clinical workflows are adequately reported in a 15-item supplementary framework.

Given the increasing prevalence of oncology RCTs evaluating AI interventions, it is important to assess whether these trial protocols adhere to the SPIRIT-AI 2020 reporting guidelines. Concordance to SPIRIT 2013 and SPIRIT-AI 2020 guidelines is essential to facilitate transparency, reproducibility, and ultimately the successful translation of AI technologies into oncology practice. However, the extent to which oncology RCTs comply with SPIRIT-AI reporting standards remains unknown.

This systematic review aims to evaluate the concordance of RCT protocols investigating AI interventions in oncology to the SPIRIT 2013 and SPIRIT-AI 2020 reporting guidelines. By critically appraising protocol completeness, this study seeks to identify gaps in protocol reporting across the current landscape of AI trial design in oncology. Understanding these gaps will inform future research efforts aimed at addressing deficiencies in reproducibility of AI research and contribute to the development of robust evidence to support the safe and effective integration of AI into oncology care.^11^

## Methods

We conducted a search of Ovid Medline and Embase on October 22, 2024, using search terms related to oncology (“neoplasm,” “cancer,” “tumor,” “onco”), clinical trials (“protocol” “trial protocol,” “study protocol,” “research protocol”), and artificial intelligence (“artificial intelligence,” “machine learning,” “deep learning,” “neural network”), in consultation with a librarian. We included English, primary, peer-reviewed research articles published from the year 2000 onwards. Non-English articles, editorials, conference abstracts, secondary reviews, and preprints were excluded. The complete search strategy is detailed in Supplementary Table S1. This systematic review adhered to the PRISMA reporting guidelines and was prospectively registered in PROSPERO (CRD42024606229).

The literature search identified 2,768 articles. After de-duplication of 905 articles, 1,863 unique articles remained for abstract screening. Abstract screening was performed in duplicate by 3 reviewers (E.H., K.P., M.C.) and resolved through discussion with a fourth reviewer (D.C.). Abstracts were included if the article described a primary, randomized clinical trial protocol involving an artificial intelligence intervention in at least one trial arm and involved screening, diagnosis or management of cancer. Articles focusing solely on classical statistical methods, secondary research, or non-oncology settings were excluded. Abstract screening resulted in 284 articles for full-text review. Full-text screening, also conducted in duplicate by the same team, screen using the same inclusion and exclusion criteria. The reference lists of the included articles were screened to ensure comprehensive coverage of relevant research.

Key attributes of the included studies were extracted in duplicate (E.H., K.P., M.C.), including the purpose of the AI intervention, the target user population, participant age groups, the hosting country, clinical specialty, sample size, recruitment sites, and qualitative summaries of methodologies and primary outcomes. SPIRIT 2013 and SPIRIT-AI 2020 assessments were conducted by 3 reviewers (E.H., K.P., M.C.), with conflicts resolved through consensus discussion with a fourth reviewer (D.C.).

The analysis of SPIRIT guideline concordance focused on the percentage of RCTs reporting each item. Subgroup analyses were conducted based on types of SPIRIT checklist items (all items, AI-specific items, non-AI items). Within each trial, concordance to SPIRIT guidelines was quantified as the percentage of reported items. The primary summary metric was median concordance to reduce the effect of outlier trials with low reporting concordance that may skew the mean concordance measure. The complete data extraction is available in Supplementary Table 2. Statistical analyses were conducted using Python 3.8.9 and scipy 1.14.1.

## Results

**Figure 1.**
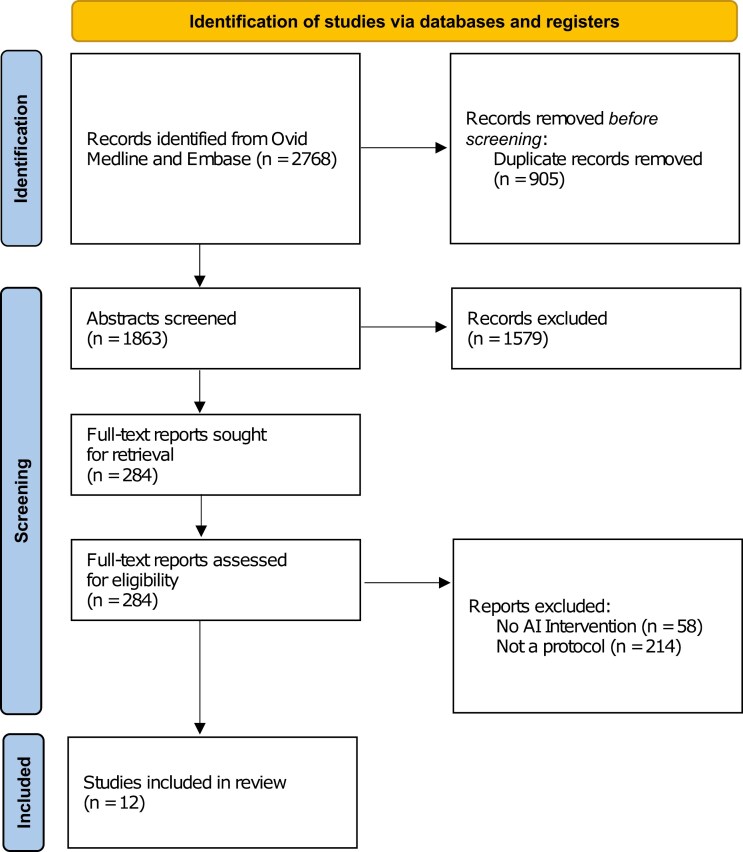
PRISMA 2020 flow diagram.

The literature search yielded 12 protocols published between 2020-2024 included in this review that met the inclusion and exclusion criteria (Figure 1).-^12-23^ Summary characteristics of the included protocols are shown in Supplementary Table S2. The majority of protocols were aimed at monitoring and supportive care (*n* = 5, 41.66%) and screening (*n* = 4, 33.33%)with intended for use of AI system by clinicians (*n* = 8, 66.67%) rather than by patients (*n* = 4, 33.33%) (Table 1). The protocols described planned studies hosted by institutions in the USA (*n* = 3, 25%) and United Kingdom (*n* = 2, 16.67%). The mean planned sample size was 3986, with the majority of studies having a sample size of over 1000 participants (*n* = 7, 58.33%). The majority of protocols planned for multiple recruitment sites (*n* = 7, 58.33%) compared to single recruitment sites (*n* = 4, 33.33%).

**Table 1.** Characteristics of included trial protocols.

Category	Type	Count	Proportion (%)
AI Purpose	Screening	4	33.33
	Monitoring or Supportive Care	5	41.67
	Diagnosis	2	16.67
	Treatment or Management	1	8.33
Intended User	Clinician	8	66.67
	Patient	4	33.33
Country	USA	3	25
	United Kingdom	2	16.67
	France	1	8.33
	Germany	1	8.33
	Netherlands	1	8.33
	China	1	8.33
	Spain	1	8.33
	South Korea	1	8.33
	Italy	1	8.33
Sample Size	>1000	7	58.33
	201-500	2	16.67
	501-1000	1	8.33
	101-200	1	8.33
	51-100	1	8.33
Recruitment Site	Single	7	58.33
	Multiple	4	33.33
	Unknown	1	8.33

The concordance of included protocols to each of the SPIRIT 2013 and SPIRIT-AI 2020 criteria is shown in Tables 2 and 3, respectively. The initial round of SPIRIT guideline scoring had a kappa score of 0.74. Across the 12 protocols, the median SPIRIT 2013 concordance was 81.92% (IQR 74.88-88.95) and the median SPIRIT-AI 2020 concordance was 78.21% (IQR 67.21-89.20).

**Table 2.** SPIRIT 2013 item-specific concordance.

SPIRIT Checklist	Concordance (*n*)	Concordance (%)
1. Descriptive title identifying the study design, population, interventions, and, if applicable, trial acronym	12	100
2a. Trial identifier and registry name. If not yet registered, name of intended registry	10	83.33
2b. All items from the World Health Organization Trial Registration Dataset	9	75
3. Date and version identifier	9	75
4. Sources and types of financial, material, and other support	11	91.67
5a. Names, affiliations, and roles of protocol contributors	11	91.67
5b. Name and contact information for the trial sponsor	8	66.67
5c. Role of study sponsor and funders, if any, in study design; collection, management, analysis, and interpretation of data; writing of the report; and the decision to submit the report for publication, including whether they will have ultimate authority over any of these activities	9	75
5d. Composition, roles, and responsibilities of the coordinating center, steering committee, endpoint adjudication committee, data management team, and other individuals or groups overseeing the trial, if applicable (see Item 21a for data monitoring committee)	11	91.67
6a. Description of research question and justification for undertaking the trial, including summary of relevant studies (published and unpublished) examining benefits and harms for each intervention	12	100
6b. Explanation for choice of comparators	12	100
7. Specific objectives or hypotheses	12	100
8. Description of trial design including type of trial (for example, parallel group, crossover, factorial, single group), allocation ratio, and framework (for example, superiority, equivalence, noninferiority, exploratory)	10	83.33
9. Description of study settings (for example, community clinic, academic hospital) and list of countries where data will be collected. Reference to where list of study sites can be obtained	11	91.67
10. Inclusion and exclusion criteria for participants. If applicable, eligibility criteria for study centers and individuals who will perform the interventions (for example, surgeons, psychotherapists)	12	100
11a. Interventions for each group with sufficient detail to allow replication, including how and when they will be administered	12	100
11b. Criteria for discontinuing or modifying allocated interventions for a given trial participant (for example, drug dose change in response to harms, participant request, or improving/worsening disease)	8	66.67
11c. Strategies to improve adherence to intervention protocols, and any procedures for monitoring adherence (for example, drug tablet return, laboratory tests)	11	91.67
11d. Relevant concomitant care and interventions that are permitted or prohibited during the trial	12	100
12. Primary, secondary, and other outcomes, including the specific measurement variable (for example, systolic blood pressure), analysis metric (for example, change from baseline, final value, time to event), method of aggregation (for example, median, proportion), and time point for each outcome. Explanation of the clinical relevance of chosen efficacy and harm outcomes is strongly recommended	12	100
13. Time schedule of enrollment, interventions (including any run-ins and washouts), assessments, and visits for participants. A schematic diagram is highly recommended.	12	100
14. Estimated number of participants needed to achieve study objectives and how it was determined, including clinical and statistical assumptions supporting any sample size calculations	12	100
15. Strategies for achieving adequate participant enrollment to reach target sample size	11	91.67
16a. Method of generating the allocation sequence (for example, computer-generated random numbers), and list of any factors for stratification. To reduce predictability of a random sequence, details of any planned restriction (for example, blocking) should be provided in a separate document that is unavailable to those who enroll participants or assign interventions	11	91.67
16b. Mechanism of implementing the allocation sequence (for example, central telephone; sequentially numbered, opaque, sealed envelopes), describing any steps to conceal the sequence until interventions are assigned	11	91.67
16c. Who will generate the allocation sequence, who will enroll participants, and who will assign participants to interventions	11	91.67
17a. Who will be blinded after assignment to interventions (for example, trial participants, care providers, outcome assessors, data analysts), and how	7	58.33
17b. If blinded, circumstances under which unblinding is permissible, and procedure for revealing a participant’s allocated intervention during the trial	3	25
18a. Plans for assessment and collection of outcome, baseline, and other trial data, including any related processes to promote data quality (for example, duplicate measurements, training of assessors) and a description of study instruments (for example, questionnaires, laboratory tests) along with their reliability and validity, if known. Reference to where data collection forms can be found, if not in the protocol	12	100
18b. Plans to promote participant retention and complete follow-up, including list of any outcome data to be collected for participants who discontinue or deviate from intervention protocols	6	50
19. Plans for data entry, coding, security, and storage, including any related processes to promote data quality (for example, double data entry; range checks for data values). Reference to where details of data management procedures can be found, if not in the protocol	11	91.67
20a. Statistical methods for analyzing primary and secondary outcomes. Reference to where other details of the statistical analysis plan can be found, if not in the protocol	12	100
20b. Methods for any additional analyses (for example, subgroup and adjusted analyses)	9	75
20c. Definition of analysis population relating to protocol non-adherence (for example, as randomized analysis), and any statistical methods to handle missing data (for example, multiple imputation)	9	75
21a. Composition of data monitoring committee (DMC); summary of its role and reporting structure; statement of whether it is independent from the sponsor and competing interests; and reference to where further details about its charter can be found, if not in the protocol. Alternatively, an explanation of why a DMC is not needed	9	75
21b. Description of any interim analyses and stopping guidelines, including who will have access to these interim results and make the final decision to terminate the trial	3	25
22. Plans for collecting, assessing, reporting, and managing solicited and spontaneously reported adverse events and other unintended effects of trial interventions or trial conduct	8	66.67
23. Frequency and procedures for auditing trial conduct, if any, and whether the process will be independent from investigators and the sponsor	8	66.67
24. Plans for seeking research ethics committee/institutional review board (REC/IRB) approval	12	100
25. Plans for communicating important protocol modifications (for example, changes to eligibility criteria, outcomes, analyses) to relevant parties (for example, investigators, REC/IRBs, trial participants, trial registries, journals, regulators)	10	83.33
26a. Who will obtain informed consent or assent from potential trial participants or authorized surrogates, and how (see Item 32)	11	91.67
26b. Additional consent provisions for collection and use of participant data and biological specimens in ancillary studies, if applicable	2	16.67
27. How personal information about potential and enrolled participants will be collected, shared, and maintained in order to protect confidentiality before, during, and after the trial	12	100
28. Financial and other competing interests for principal investigators for the overall trial and each study site	12	100
29. Statement of who will have access to the final trial dataset, and disclosure of contractual agreements that limit such access for investigators	11	91.67
30. Provisions, if any, for ancillary and post-trial care, and for compensation to those who suffer harm from trial participation	5	41.67
31a. Plans for investigators and sponsor to communicate trial results to participants, healthcare professionals, the public, and other relevant groups (for example, via publication, reporting in results databases, or other data sharing arrangements), including any publication restrictions	11	91.67
31b. Authorship eligibility guidelines and any intended use of professional writers	11	91.67
31c. Plans, if any, for granting public access to the full protocol, participant-level dataset, and statistical code	12	100
32. Model consent form and other related documentation given to participants and authorized surrogates	9	75
33. Plans for collection, laboratory evaluation, and storage of biological specimens for genetic or molecular analysis in the current trial and for future use in ancillary studies, if applicable	0	0

**Table 3.** SPIRIT-AI 2020 item-specific concordance.

SPIRIT-AI Checklist	Concordance (*n*)	Concordance (%)
1-i. Indicate that the intervention involves artificial intelligence/machine learning and specify the type of model.	12	100
1-ii. Specify the intended use of the AI intervention.	11	91.67
6a-i. Explain the intended use of the AI intervention in the context of the clinical pathway, including its purpose and its intended users (for example, healthcare professionals, patients, public).	12	100
6a-ii. Describe any pre-existing evidence for the AI intervention.	11	91.67
9-i. Describe the onsite and offsite requirements needed to integrate the AI intervention into the trial setting.	9	75
10-i. State the inclusion and exclusion criteria at the level of participants.	12	100
10-ii. State the inclusion and exclusion criteria at the level of the input data.	6	50
11a-i. State which version of the AI algorithm will be used.	8	66.67
11a-ii. Specify the procedure for acquiring and selecting the input data for the AI intervention.	12	100
11a-iii. Specify the procedure for assessing and handling poor-quality or unavailable input data.	7	58.33
11a-iv. Specify whether there is human-AI interaction in the handling of the input data, and what level of expertise is required for users.	11	91.67
11a-v. Specify the output of the AI intervention.	12	100
11a-vi. Explain the procedure for how the AI intervention’s output will contribute to decision-making or other elements of clinical practice.	12	100
22-i. Specify any plans to identify and analyze performance errors. If there are no plans for this, justify why not.	6	50
29-i. State whether and how the AI intervention and/or its code can be accessed, including any restrictions to access or re-use.	5	41.67

Among 51 items in the SPIRIT 2013 criteria, items related to descriptive title (Item 1), research question, motivation, and comparators (Item 6a, 6b), methodology (Item 10, 11a, 12, 13, 14), data assessment (Item 18a, 20a), and ethics, privacy, and transparency (Item 24, 27, 28, 31c) had 100% concordance across all 12 protocols (Table 2). Items related to modifying interventions for trial participants (Item 11b, 66.67%), blinding (Item 17a, 58.33%; 17b, 25%), participant retention (Item 18b, 50%), interim analysis (Item 21b, 25%), consent for ancillary studies (Item 26b, 16.67%), post-trial care (Item 30, 41.67%) and plan for collection and storage of biological specimens for molecular analysis (Item 33, 0%) were poorly concordant. No protocols reported 100% concordance to the SPIRIT 2013 guidelines.

Among 15 items in the SPIRIT-AI 2020 criteria, the indication that the intervention involves an AI component (Item 1-i), explanation of the intended use of the AI intervention (Item 6a-i), procedure for data collection (Item 11a-ii), AI intervention output (Item 11a-v), and contribution of AI intervention output to decision-making (Item 11a-vi) had 100% concordance across all 12 protocols (Table 3). The specification of the intended use of the AI intervention (Item 1-ii), description of pre-existing evidence for the AI intervention (Item 6a-ii), and description of human-AI interaction for handling of input data were observed in 11 protocols with a concordance rate of 91.67%. Lower concordance was observed for items including inclusion and exclusion of input data (Item 10-ii. 50%), handling of poor quality data (Item 11a-iii, 58.33%), analysis of performance errors (Item 22-i, 50%), and access to AI intervention and code (Item 29-i, 41.67%). Three protocols (25%) reported 100% concordance to the SPIRIT-AI 2020 guidelines.

## Discussion

The findings of this systematic review highlight promising trends and gaps in the reporting of randomized controlled trial protocols involving artificial intelligence in oncology. While the median concordance rates of 81.92% for SPIRIT 2013 and 78.21% for SPIRIT-AI 2020 guidelines suggest a high level of concordance to standard protocol elements, important deficiencies remain in areas critical to the rigorous and transparent evaluation of AI-driven interventions. Despite the potential of AI interventions to revolutionize oncology by offering advanced diagnostic, prognostic, and therapeutic decision-support systems,^24^ their adoption in clinical practice depends largely on the transparency and reproducibility of the underlying RCT protocols and associated reports.^25^

Gryaznov et al found that the median reporting concordance of general RCT protocols approved by Swiss, German, and Canadian research ethics committees was 72% (IQR, 63%-79%) in 2012 before the release of SPIRIT 2013 (*n* = 257) and 77% (IQR, 68%-82%) in 2016 after the release of SPIRIT 2013 (*n* = 292).^26^ Likewise, we observed a comparable median SPIRIT 2013 concordance of 81.92% (IQR 74.88-88.95), which suggests that RCT protocols may still face gaps in standardized reporting. Holzwanger et al. conducted a review of SPIRIT-AI concordance among AI clinical trial protocols in gastroenterology in 2021 that found a mean concordance of 95.62%, with 5 out of 8 studies scoring 100% in concordance.^27^ Surprisingly, we observed lower average concordance which may suggest that broad awareness and concordance to reporting guidelines, especially among more recent studies included in our review, remains a concern that remains to be wholly addressed. However, we note that the low sample size of published protocols across reviews of SPIRIT-AI concordance may underpower comparisons between protocols of AI RCTs across different domains. There are very few reviews assessing SPIRIT-AI concordance, highlighting the need for such evaluations to draw awareness to the specific reporting gaps that can improve overall reporting guideline adherence.

The open-access publication of trial protocols in parallel with trial results should be encouraged to promote critical peer-reviewed evaluation of study design and research reproducibility. For example, a 2021 review of CONSORT-AI concordance of clinical trials involving artificial intelligence found that out of 29 trial reports included in the review, only 3 associated trial protocols could be retrieved.^28^ Likewise, a 2024 review of SPIRIT and CONSORT concordance of pediatric neuro-oncology protocols and trials included 9 trial protocols compared to 76 phase II and III trial reports.^29^ Publishing trial protocols with trial reports enhances transparency, allows for assessment of concordance to planned trial methods, and helps identify potential deviations from protocols that may contribute to potential biases or poor outcomes.

Our findings indicate a strong concordance of trial protocols to reporting critical trial elements such as descriptive titles (Item 1), research questions (Item 6a, 6b, 7), and methodological design (Item 10, 11a, 11d, 12, 13, 14) which are essential for establishing trial validity across studies. However, poor concordance in areas such as blinding (Item 17a, 17b), participant retention (Item 18b), interim analyses (Item 21b), and harm management strategies (Item 11b, 30) raises concerns about the methodological reproducibility and generalizability of AI-related trial outcomes. Ethical transparency and robust data governance are pivotal in AI-driven oncology trials due to the complexity of data collection, processing, and analysis. Notably, preventive planning and management of potential harm of AI intervention, in accordance with the “do no harm” principle of medical ethics, is an important limitation of current trial reporting in AI oncology trials.^30^ Despite the purported advances in clinical decision making offered by supportive AI interventions based on statistical performance metrics, the complexity of these “black box” algorithms may introduce unintended biases that lead to patient harm, and recent examples include underdiagnosis of chronic kidney disease,^31^ poor prediction of health care costs associated with health needs,^32^ and propagation of healthcare stereotypes^33^ based on race. Thus, AI interventions evaluated using RCTs should not only demonstrate improvement in patient-reported outcomes or clinical outcomes but also be safely tested in environments with effective harm management strategies.^34^

Despite full compliance with SPIRIT criteria related to ethics (Item 24) and privacy (Item 27) during the planned trial duration, gaps were observed in consent for ancillary studies (Item 26b) and post-trial care (Item 30). The latter is particularly concerning, as post-trial care often extends beyond arranging access to the trial intervention, but also requires responsible transitioning of participants with appropriate clinical care and social services following trial conclusion.^35^ In addition, ancillary analyses often provide valuable insights into AI performance across diverse patient subgroup analyses, but require additional context-dependent confirmation of participant consent for reuse of different types of specimens and data.^36^ Study investigators should plan ahead in trial design to consent participants for the primary trial and specific indications for reuse of data in ancillary studies. Lastly, the lack of compliance with specimen collection and storage for molecular analysis underscores the need for comprehensive data management plans that align with evolving ethical frameworks and regulatory requirements for molecular oncology research coupled with emergent AI interventions.^37,38^ However, we note the reporting of this SPIRIT-AI guideline item may not be broadly applicable since not all studies involve molecular analysis of specimens.

The inclusion of AI-specific criteria in the SPIRIT-AI 2020 guidelines represents an important step toward ensuring the responsible integration of AI interventions in trials. Encouragingly, all protocols reported AI-specific components such as the intended use of the intervention (item 6a-i), data collection procedures (Item 11a-ii), and AI output contributions to clinical decision-making (Item 11a-v, 11a-vi). Explicit indication of the input data types and format used to inform the AI intervention prediction allows for assessment of data quality, as poor quality and missing data can compromise the quality of AI systems.^39^ However, we observed that despite reporting of data collection procedures in protocols of AI oncology trials, there was poor concordance of reporting the procedure for assessment of data quality and availability (Item 11a-iii). Specifying these details in clinical trial protocols ensures that the clinical AI system is trained and evaluated on data representative of its intended use, thereby enhancing the reliability of the trial outcomes and clinical translation of AI-based findings whose performance is heavily dependent on data quality.

Moreover, we observed that all protocols reported inclusion and exclusion criteria of participants (Item 10-i) but often failed to report inclusion and exclusion criteria of input data (Item 10-ii) to the AI intervention. Importantly, input data of AI interventions often include data features that collectively describe participants enrolled in the trial.^40^ Without clear reporting criteria of inclusion and exclusion of both patients and patient-derived input data, the AI system may process data that is not representative of the target patient population, leading to biased or unreliable outcomes.^41^ This omission hampers the reproducibility of the study and complicates the assessment of the AI intervention’s performance across external clinical settings. Moreover, it challenges the integration of the AI tool into clinical practice, as stakeholders may lack important information about the data characteristics that influence the system’s behavior.

Finally, we observed poor reporting on proposed analyses of performance errors (Item 22-i) necessary to conduct quality improvement of the AI intervention, which is especially important in scenarios where the intervention may fail to meet standards of clinical care.^42^ External validation of AI interventions remains a necessary step towards fostering clinical trust and adoption across diverse clinical settings, ensuring that both performance metrics and patient outcomes are positively impacted by the introduction of the AI intervention.^43^

Given that only 5 out of 12 (41.67%) of included protocols in this review reported information about access to the AI intervention or code, poor reporting of access to the AI intervention code (item 29-i) presents a challenge for external validation of the AI system’s performance. Promotion of open science may serve as one step towards addressing the emergent reproducibility crisis in AI research by allowing both scientists and clinicians to independently assess the reliability and applicability of the AI intervention in different settings.^44^ This transparency also fosters improved peer collaboration, where community-driven feedback can accelerate refinements and innovations in AI methodologies. Notable trends in open-source communities, exemplified by platforms such as GitHub and AI model hubs like Hugging Face and TensorFlow Model Garden, have facilitated the widespread dissemination of code and implementation details. Although suboptimal reporting of data access was observed in our review, we highlight how the subset protocols that reported open data access can set a promising precedent and model for how data access can be wholly reported in future AI RCT protocols.

Addressing these gaps will require concerted efforts from regulatory bodies, funding agencies, and journal editorial boards to mandate comprehensive reporting that ensures full transparency. To bridge existing gaps, future oncology trials incorporating AI should prioritize strategies such as enhanced blinding and bias mitigation, comprehensive data stewardship, and algorithmic transparency. Improved reporting on blinding mechanisms and participant retention strategies can help mitigate biases and improve the generalizability of trial results. Adoption of standardized protocols for data collection, storage, and sharing coupled with consistent protocol reporting will ensure reproducibility and compliance with regulatory and ethical standards. Open access to AI models and performance metrics is crucial to foster reproducibility and facilitate independent validation. Additionally, educational initiatives for trial engineers and clinicians can enhance familiarity with SPIRIT-AI guidelines and AI-specific reporting requirements. One way to ensure adherence to the SPIRIT-AI checklist is to mandate that journals require a statement from authors that the protocol that they used for the study and the study report both adhere to the checklist as outlined in the SPIRIT-AI guidelines. This will go a long way to promote adherence to these important guidelines for studies that use AI in their design. While AI holds transformative potential in oncology, our review underscores the critical need for improved transparency and concordance with established trial protocol reporting guidelines. Concordance to both SPIRIT-AI and CONSORT-AI reporting guidelines for RCT protocols and reports provides a framework for transparent reporting of the entire lifecycle of AI-driven clinical research.^10,45^ SPIRIT-AI ensures rigorous planning and transparency in trial design, addressing potential biases and uncertainties early on, while CONSORT-AI focuses on clear and comprehensive reporting of completed trials, enabling reproducibility and critical evaluation.

Researchers can take targeted steps to address specific gaps in AI research reporting. For instance, to improve reporting of blinding procedures, trial teams should develop and implement standardized, open-access training protocols for both clinical staff and evaluators, along with independent audits to verify the integrity of blinding throughout the study. To improve data quality management, establishing multi-tiered validation processes for quality checks, real-time monitoring systems for data input and output, and dedicated data stewardship roles can help promote data consistency and reliability. It is also important to recognize that different AI applications may require varying degrees of adherence to these guidelines; for example, diagnostic imaging and personalized treatment algorithms might necessitate more stringent blinding and data management and security protocols compared to AI applications aimed at administrative or clinical workflow optimization. Tailoring these recommendations to the specific context of the AI intervention will not only align with SPIRIT-AI and CONSORT-AI research guidelines but also enhance the overall reproducibility of the research necessary to promote their safe and effective integration into clinical practice.

Taken together, the advent of reporting guidelines has contributed to improved completeness of research reporting, particularly in how researchers manage and report critical components such as input data handling, data quality assessment, and performance error analysis. Martindale et al.(2024) found that RCT reports that reported usage of CONSORT-AI guidelines had higher median concordance to their respective guideline than RCT reports that reported usage of CONSORT guidelines or did not use any guidelines.^46^ These results suggest that as AI research in RCT settings continues to become more prevalent, increasing the adoption of AI-specific reporting guidelines can help ensure that AI-specific elements of research methodology and results are wholly reported. Despite these advancements, certain core challenges remain, such as balancing model complexity with interpretability, underscoring both the progress made and the areas of reporting in need of further refinement. Given the unique components of AI interventions as outlined in the CONSORT-AI and SPIRIT-AI reporting guidelines for RCT reports and RCT protocols respectively, this shift towards focused AI intervention-specific reporting highlights a promising scientific commitment to transparency and reproducibility in AI trial design.

This systematic review has several limitations. First, the small sample size of 12 included protocols limits the generalizability of our findings and may not fully represent the broader landscape of AI-driven trial protocols across other clinician domains beyond oncology. The small sample size limited the ability to conduct temporal and subgroup analyses. Second, the inclusion of protocols of AI RCTs in oncology may fail to capture protocols of other prospective study types that may be useful to characterize the landscape of protocol reporting concordance. For example, a recently published review of clinical trials evaluating AI interventions in oncology found 536 trials registered on clinicaltrials.gov in December 2023, of which 112 were confirmed to be RCTs.^47^ Third, the inclusion of peer-reviewed primary research articles led to the exclusion of preprints and grey literature, which may contain emerging trial designs and methodologies. Fourth, trial reports often do not publish their associated trial protocols, and when they do, the published trial protocols may be more concordant to reporting guidelines due to peer review and journal editorial practices.

## Conclusion

The integration of AI into oncology holds immense potential to revolutionize cancer care by improving early detection, diagnosis, and personalized treatment strategies. Our systematic review highlights that while the concordance of AI RCT protocols in oncology to the SPIRIT 2013 and SPIRIT-AI 2020 guidelines is moderately high, critical gaps remain in key areas such as data quality reporting, blinding, harm management strategies, and post-trial care. These deficiencies underscore the need for improved transparency and methodological rigor in AI-driven oncology trials to ensure their reproducibility, ethical integrity, and clinical utility.

To bridge existing gaps, protocols of oncology RCTs involving AI interventions should prioritize comprehensive reporting of AI system development and validation, data quality assurance, and ethical considerations such as post-trial patient care and informed consent for ancillary studies. Encouraging open access to AI models, algorithms, and datasets will be essential to foster reproducibility and external validation. Moreover, enhanced educational and awareness initiatives for trial investigators, regulatory bodies, funding agencies, and journal editorial boards can improve concordance with reporting guidelines, ensuring that AI interventions in oncology are rigorously evaluated and safely integrated into clinical practice.

## Supplementary Material

oyaf112_suppl_Supplementary_Tables_1

oyaf112_suppl_Supplementary_Tables_2

## Data Availability

All data reported in this study is available in the main figures, tables, and supplementary tables. Additional information is available upon reasonable request to the corresponding author.
