# Supplementary material for: Concordance with SPIRIT-AI guidelines in reporting of randomized controlled trial protocols investigating artificial intelligence in oncology: a systematic review
Source: Oncologist. 2025 May 27;30(5):oyaf112. doi: 10.1093/oncolo/oyaf112 (PMC12107541; doi:10.1093/oncolo/oyaf112)
Supplement: oyaf112_suppl_Supplementary_Tables_1 [file oyaf112_suppl_supplementary_tables_1.docx]

**Supplementary Table 1**. Full search strategy used for OVID MEDLINE and Embase.

| **Set** | **Search Statement** |
| --- | --- |
| 1 | artificial intelligence/ |
| 2 | (artificial adj2 intelligence).mp. |
| 3 | machine learning/ |
| 4 | (machine adj2 learning).mp. |
| 5 | deep learning/ |
| 6 | (deep adj2 learn*).mp. |
| 7 | supervised machine learning/ |
| 8 | (supervised learning adj2 unsupervised learning).mp. |
| 9 | neural networks, computer/ |
| 10 | (computer adj2 neural adj2 network*).mp. |
| 11 | neural network*.mp. |
| 12 | or/1-11 |
| 13 | neoplas*.tw,kf. |
| 14 | cancer*.tw,kf. |
| 15 | tumo?r*.tw,kf. |
| 16 | onco*.tw,kf. |
| 17 | or/13-16 |
| 18 | protocol*.tw,kf. |
| 19 | (trial adj2 protocol).mp. |
| 20 | (study adj2 protocol).mp. |
| 21 | (research adj2 protocol).mp. |
| 22 | or/18-21 |
| 23 | 12 and 17 and 22 |
| 24 | (exp animal/ not (exp animals/ and exp humans/)) or ((exp animals/ or exp animalexperiment/) not humans/) or (in vitro or in vivo or cell lines).ti. |
| 25 | 21 not 22 |
| 26 | limit 25 to yr="2000 -Current" |
| 27 | limit 26 to english language |
